# Postmortem diagnostics in sudden unexpected death in infants and children: use and utility

**DOI:** 10.1007/s00431-025-06035-6

**Published:** 2025-03-05

**Authors:** A. M. Pries, A. C. van der Gugten, H. A. Moll, W. M. Klein, PESUDIC Collaborative, A. Custers, E. Edelenbos, J. Fuijkschot, B. Levelink, P. J. Puiman, J. M. Ruskamp, B. Semmekrot, K. T. Verbruggen, H. Vlaardingerbroek, J. Fuijkschot, P. J. Puiman

**Affiliations:** 1 Department of General Paediatrics, Erasmus University Medical Center Sophia Children’s Hospital, Rotterdam, the Netherlands; 2 Department of Paediatrics, University Medical Center Utrecht Wilhelmina Children’s Hospital, Utrecht, the Netherlands; 3 Department of Radiology, Radboud University Medical Center Amalia Children’s Hospital, Nijmegen, the Netherlands; 4 Department of Paediatrics, Radboud University Medical Center Amalia Children’s Hospital, Nijmegen, the Netherlands

**Keywords:** Child death review, Forensic, SUDI, SIDS, SDY, SUDC

## Abstract

Sudden unexpected death in infants (SUDI) and children (SUDC) requires thorough investigation to identify causes and prevention strategies. In the Netherlands, these deaths are investigated using the standardized postmortem evaluation of sudden unexpected death in infants and children (PESUDIC) procedure. This study examines the use of various diagnostic tests within PESUDIC and their effectiveness in determining causes of death. This observational study included infants and children who died suddenly and underwent the PESUDIC procedure from 2016 to 2022. Standardized data on medical history, postmortem examinations, and diagnostic outcomes were collected. Findings were classified by consensus of two experts as “contributory” if they supported the cause of death and “decisive” if they were leading for determination. A total of 275 cases were included. Median age was 13 months (IQR 3.5–73.3). Fifty-nine percent were boys. Over 95% of cases had a medical history, postmortem physical examination, biochemical, and microbiological testing available. Total body postmortem CT and/or MRI was done in 93% (*n* = 255) and autopsy in 62% (*n* = 171). The cause of death was determined in 193 (70%). History, imaging, and autopsy provided contributory results in 50% (*n* = 137/275), 40% (*n* = 103/255), and 67% (*n* = 115/171) of applicable cases, respectively. More than two different tests showed contributory findings in 52% of diagnosed cases. Autopsy and microbiological testing had decisive findings most often: in 83/171 and 44/265 cases respectively. *Conclusion*: A routinely performed wide array of postmortem investigations has additional value to an autopsy for identifying the cause of death in SUDI and SUDC. A thorough SUDY investigation should therefore minimally include an autopsy, microbiological testing, and whole-body imaging.

**Table:** 

**What is Known:** *• A thorough postmortem investigation into the cause of sudden death in infants and children can provide an explanation of the death and identify potential preventable causes.* **What is New:** *• A thorough postmortem investigation for sudden unexpected death in infants and children should minimally include an autopsy, microbiological testing and whole-body imaging.*

**What is Known:**

*• A thorough postmortem investigation into the cause of sudden death in infants and children can provide an explanation of the death and identify potential preventable causes.*

**What is New:**

*• A thorough postmortem investigation for sudden unexpected death in infants and children should minimally include an autopsy, microbiological testing and whole-body imaging.*

## Introduction

Sudden unexpected death in children is the term for the sudden and initially unexplained, though presumed natural, death of an individual 1–18 years. Sudden unexpected death in infancy (SUDI) is one of the most common diagnoses for deceased infants (< 12 months of age) in Western Europe [1]. Since sudden unexpected death in older children is not recorded in the International Classification of Diseases, the exact prevalence in Europe is not known, but it occurs less frequently than SUDI [2]. The sudden death of a child from an unclear cause has a large impact. Both family members and involved health professionals are left with many unanswered questions. In these cases, a thorough postmortem investigation into the cause of death can provide an explanation and identify potential preventable causes [3, 4]. However, what encompasses a thorough postmortem investigation is still up for debate.

In the Netherlands, SUDI and sudden unexpected death in children (SUDC) are estimated to occur in 50 children every year [5]. To provide a cause of death, these cases can be investigated through the Dutch Postmortem Evaluation of Sudden Unexpected Death in Infants and Children (PESUDIC). This procedure consists of an extensive step-wise diagnostic protocol including a comprehensive history, physical examination, biochemical analysis, microbiological testing, radiological imaging, autopsy, and finally a multidisciplinary panel discussion to determine the cause of death in consensus [6]. Cases with signs of an unnatural cause are excluded from participating in the PESUDIC procedure. The PESUDIC procedure is not mandatory, meaning parents have to consent to the different steps in the procedure. In 58% of the children that were investigated through the PESUDIC procedure between 2016 and 2021, the cause of death was explained, and in an additional 13%, a plausible cause was identified [6]. The value of the different diagnostic investigations for the PESUDIC procedure has not been evaluated yet.

Conventional autopsy performed by a trained pediatric pathologist is considered the gold standard for identifying the cause of death. In recent years, other diagnostic tests such as postmortem whole-body magnetic resonance imaging (MRI) and computed tomography (CT) have been increasingly used for investigating sudden child deaths [7–11]. These radiological methods have the great advantage of being non-invasive. Multiple studies have been conducted on the accuracy of imaging for identifying the cause of death in SUDI and SUDC [12–16]. These studies were performed in small groups ranging from 11 to 54 children, and their results are mixed, showing an accordance with autopsy results ranging between 18 and 83%.

Little is known about the utility of postmortem chemical laboratory tests or microbiological testing in SUDI or SUDC cases. Two studies found no additional benefit from microbiological testing before autopsy in infants with sudden death [17, 18]. However, bacteriological cultures taken during autopsy were often decisive for the diagnosis in sudden death cases [17–19]. Virological investigations seem to yield low results [20, 21]. No reports on biochemical laboratory testing for sudden death in children are reported.

The aim of this national observational study is to describe the use of the different postmortem diagnostic tests in the standardized PESUDIC procedure and study their diagnostic value for determining the cause of death in sudden unexpected death in infants and children.

## Methods

This study has a retrospective observational design and analyses data collected on participants of the PESUDIC procedure. The design and outcomes of the PESUDIC procedure were reported in a previous article [6]. Since PESUDIC deaths are considered natural by the forensic medical examiner, parental consent is required for the different steps of the procedure. The study was exempted from review by the Medical Research Ethics Committee of the Erasmus University Medical Centre Rotterdam (reference number MEC-2021–0421), and the need for informed consent has been waived.

### Study population

Infants (< 12 months) and children (1–18 years) were included if they were investigated through the PESUDIC procedure, and parents did not object to the use of their data for research purposes [6]. For the PESUDIC procedure, Dutch infants (< 12 months) and children (1–18 years) are eligible if the forensic medical examiner deems their death unexpected and natural but without an immediately apparent cause such as a known chronic lethal disease or exterior signs of an acute illness (unexplained). Death is considered unexpected and unexplained if the child is presumed to be previously in good health, in a stable condition of chronic disease, or experiencing mild acute illness not expected to cause death. No time restriction for the start of possible symptoms is used. Parents need to consent to at least one element of the procedure. Cases of stillbirth, peripartum death, or children who had not yet been discharged home after birth are not eligible for the PESUDIC procedure.

### Data collection and analysis

Detailed and standardized data on the medical history, postmortem physical examination, and diagnostic outcomes of the PESUDIC procedure were documented in the local electronic patient health records of all Dutch University Medical Centers. A Castor database (Ciwit BV. Castor Electronic Data Capture. 2016. Version 2024.2.4.1, Amsterdam) was constructed and focused on the diagnostic procedures and outcomes using standardized forms. Diagnostic procedures were performed according to the national PESUDIC protocol, which includes imaging and autopsy guidelines. The postmortem investigations were categorized into types of tests, and the main six were identified: history, physical examination, biochemical testing (blood, spinal fluid, vitreous humor, urine, hair), microbiological testing (blood, spinal fluid, throat/nasopharynx swab, feces/rectum swab, urine, skin), radiological imaging (whole-body CT and/or MRI scan), and autopsy (with or without the brain). Further diagnostic tests performed through the PESUDIC procedure were metabolic and genetic testing, which were not used routinely. Data were collected from the first PESUDIC case in October 2016 until December 2022. For data analysis, SPSS 28 for Windows (IBM Corp. Released 2021. IBM SPSS Statistics for Windows, Version 28.0. Armonk, NY: IBM Corp) was used. The data were summarized with descriptive analyses and analyzed using chi-square tests and an alpha level of 0.05.

### Outcome measures

Comorbidity was defined according to the Pediatric Medical Complexity Algorithm, also prematurity and/or birthweight small for gestational age were defined as comorbidity [22]. Conclusions on the cause of death were made in a multidisciplinary audit as part of the PESUDIC procedure; this was the gold standard. A cause of death was categorized as explained, plausible, or unexplained. An explained cause had an abnormal postmortem finding corroborating with the history or a full explanation of the death provided by an indisputable postmortem finding. A cause was considered plausible in cases where a postmortem finding did not fit with the history or vice versa. The cause of death remained unexplained in cases with an absence of history and postmortem findings related to a lethal condition. Discrepancies on the category of the cause of death were discussed by the first author (A.M.P) and the pediatrician managing the PESUDIC case until consensus was reached [6]. For the descriptions in this study, the explained and plausible categories were grouped as having a determined cause of death. If age categories were used, cases were divided into age groups of < 1 year, 1–6 years, 6–12 years, and > 12 years.

The utility of the different diagnostic tests in cases with determined causes of death was independently judged by two authors (A.M.P and P.J.P, J.F or H.M), and discrepancies were decided on by a third author (H.M or P.J.P). Findings related to postmortem changes or life-saving procedures were regarded as negative findings. Diagnostic investigations were considered contributory if their results corroborated the cause of death. A blood C-reactive protein (CRP) level over 60 mg/l was deemed contributory in cases with a bacterial infection as the cause of death [23]. In cases with both a positive culture in blood and spinal fluid, the culture in spinal fluid was deemed decisive over the blood culture because of its better protection against contamination, unless the pathogen primarily causes sepsis or the cause of death was categorized as primarily sepsis. If a plethora of different pathogens was found with microbiological testing, it was not deemed contributory. The one test that was leading for determining the underlying cause of death out of all contributory tests was deemed decisive. If multiple diagnostic tests provided the cause of death, the least invasive or elaborate test was deemed as the decisive one.

## Results

**Fig. 1:**
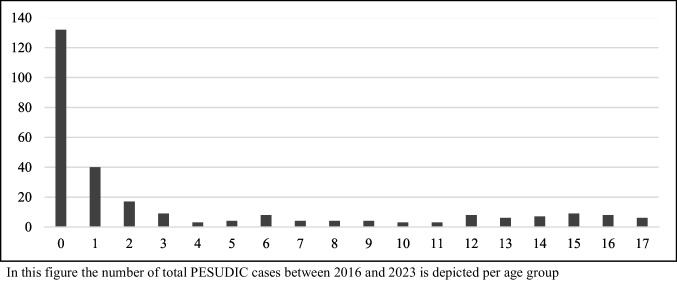
Distribution of age in years in study population

In total, 275 cases were included. Among these, 59% were male and the median age was 13.0 months (IQR 3.5–73.3). Most children became critically ill needing immediate resuscitation at home (*n* = 204, 74%) of which 139 (64%) also died at home. Of all cases, 68% (*n* = 188) died unwitnessed and unsupervised, mostly during the night. Comorbidity was present in 38% (*n* = 104) of cases. In 37% (*n* = 101) of cases, the caregivers had sought medical help for symptoms their child was experiencing before their death. Table 1 shows the basic characteristics, and Fig. 1 shows the age distribution of the study population. In 193 of the 275 cases (70%), a cause of death was determined through the PESUDIC procedure. Of these, 171 causes were categorized as explained and 22 as plausible.

**Table 1:** Basic characteristics of included cases

	Cases *N* = 275 (% of total cases)
Sex—male	161 (59)
Age—median (months)	13.0 (IQR 3.5–73.3)
Comorbidity	104 (38)
Scene of collapse	
Home	204 (74)
Hospital	26 (10)
Home family/friends	12 (4)
Other	33 (12)
Place of death	
Home	139 (51)
Hospital	111 (40)
Home family/friends	9 (3)
Other	16 (6)
Medical help-seeking for symptoms	101 (37)
Collapse during unsupervised/sleep period	188 (68)
Resuscitation attempt by professionals	238 (87)

A medical history, postmortem physical examination, and microbiological testing were performed in over 95% of cases (Table 2). Biochemical analysis was done in 92% (*n* = 252), and a postmortem CT and/or MRI was performed in 93% (*n* = 255). An autopsy was performed in 62% (*n* = 171) of cases and included the brain in 48% (*n* = 82).

**Table 2:** Use and yield of diagnostic work-up investigations

	Performed *N* (% total cases)	Contributive *N* (% performed)	Decisive^b^ *N* (% performed)
History	275 (100)	137 (50)	9 (3)
Physical examination	260 (95)	32 (12)	5 (2)
Biochemical tests	252 (92)	35 (14)	5 (2)
Blood	184 (67)	30 (16)	4 (2)
Spinal fluid	188 (68)	4 (2)	-
Vitreous humor	79 (29)	5 (6)	1 (1)
Urine	89 (32)	2 (2)	-
Hair	12 (4)	-	-
Microbiological tests	265 (96)	81 (31)	44 (17)
Blood	245 (89)	56 (23)	21 (9)
Spinal fluid	204 (74)	30 (15)	15 (7)
Throat/nasopharynx	254 (92)	56 (22)	-
Feces/rectum swab	172 (63)	19 (7)	7 (4)
Urine	118 (43)	6 (5)	-
Skin	42 (15)	3 (7)	1 (2)
Imaging^a^	255 (93)	103 (40)	39 (15)
CT scan	128 (47)	56 (44)	11 (9)
MRI scan	191 (70)	72 (38)	28 (15)
Autopsy	171 (62)	115 (67)	83 (49)
Including brain	82 (30)	50 (61)	33 (40)

^a^64 children had both a CT scan and MRI scan performed and in 25 both scans had contributive findings. ^b^In 2 cases, metabolic testing was decisive and in 6 genetic testing

The history provided contributive information in 137 of 275 cases (50%) and was decisive for 9 cases (3%), see Table 2. The physical examination and biochemical analysis were least frequently contributive and/or decisive. On the other hand, in 81 of 265 cases where microbiological testing was performed (31%), findings were contributive, and in 44 (17%), findings were decisive. Contributive microbiological findings could be found at all sites: blood (*n* = 56), spinal fluid (*n* = 30), throat or nasopharynx (*n* = 56), feces (*n* = 19), urine (*n* = 5), and skin (*n* = 7). However, decisive microbiological findings only resulted from tests on blood (*n* = 21), spinal fluid (*n* = 15), feces (*n* = 7), and skin (*n* = 1). Postmortem radiological imaging resulted in contributive findings for 103 of 255 cases (40%) and in decisive findings for 39 cases (15%). Compared to MRI, CT scans were more often contributive (*n* = 56/128 vs. *n* = 72/191) and less often decisive (*n* = 11/128 vs. *n* = 28/191). Finally, autopsy showed contributive findings for 115 out of 171 cases (67%). An autopsy was most often decisive for determining the cause of death out of all diagnostic tests, namely, in 83 of 171 (49%) of autopsied cases. Among all 193 cases with a determined cause of death, 67 (35%) did not have an autopsy performed.

**Fig. 2:**
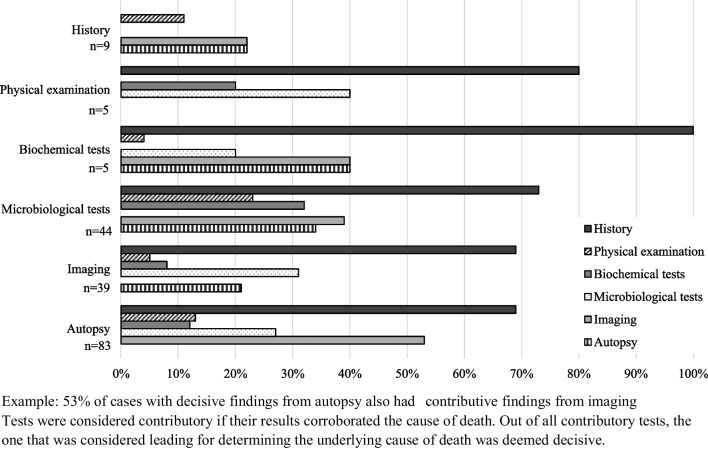
Percentage of concurrent contributive findings from other diagnostic tests in cases with decisive finding from specific tests

Cases that were diagnosed with a cause of death had contributive findings on more than two different tests in 52%. Figure 2 shows the percentages of cases with decisive findings that also had contributive findings on the other diagnostic tests per diagnostic test. For all tests, determined cases had contributive clues in the history in 69–100%. Cases diagnosed on decisive findings from physical examination did not have contributive findings from imaging or autopsy. Cases that were diagnosed based on microbiological testing results had contributive findings from imaging in 39% and from autopsy in 34%. Concurrent contributive findings for cases with decisive findings from imaging were present in 31% from microbiological testing and 21% from autopsy. Alternatively, in cases with decisive findings at autopsy, 53% had contributive findings on imaging. Concurrent contributive findings from microbiological testing were present in 27% of cases with decisive autopsy findings. In 32 cases, the cause of death was determined on findings from one single investigation: history (*n* = 7), physical examination (*n* = 1), microbiological testing (*n* = 6), genetic testing (*n* = 3), MRI (*n* = 4), and autopsy (*n* = 11).

Overall, diagnostics were used at similar rates across the age groups (Table 3). Chi-square tests comparing contributive findings for history, physical examination, biochemical analysis, microbiological testing, imaging, and autopsy between cases under 1-year-old and older children revealed that infants were significantly less likely to have contributive findings on all diagnostic tests except biochemical testing. This resulted in a significantly lower rate of causes of death determined for infants (< 12 months), namely, 52% (*n* = 69/132) compared to 87% (*n* = 124/143) for children (1–18 years). Furthermore, microbiological testing yielded contributive findings more often in children between 1 and 12 years old compared to both younger and older children. The use and yield of the diagnostics are presented according to grouped causes of death in Table 4. Cardiovascular, respiratory, and gastrointestinal causes had high rates of decisive findings at autopsy of, respectively, 86%, 100%, and 59%. Genetic testing was most often performed in cases ultimately diagnosed with a cardiovascular cause of death, namely, in 32 out of 44 cases. Decisive genetic findings were identified in 16% of these, which was the highest yield out of all types of causes of death. Microbiological testing was only decisive for diagnosing sepsis and neurological or gastrointestinal infections. Contributive findings on physical examination were not found in respiratory cases and rarely in cases with a cardiovascular type of cause of death.

**Table 3:** Use and yield of different diagnostics according to age in 275 PESUDIC cases

	Performed *N* (% total cases)				Contributive *N* (% of performed)				Decisive *N* (% of performed)			
	< 1 year	1–6 years	6–12 years	≥ 12 years	< 1 year	1–6 years	6–12 years	≥ 12 years	< 1 year	1–6 years	6–12 years	≥ 12 years
History	132 (100)	73 (100)	26 (100)	44 (100)	41 (31)^*^	47 (64)	20 (77)	29 (66)	2 (2)	1 (1)	3 (12)	3 (7)
Physical exam	126 (95)	68 (93)	25 (96)	41 (93)	9 (7)^*^	15 (22)	4 (16)	4 (10)	1 (1)	2 (3)	1 (4)	1 (2)
Fundoscopy	16 (12)	6 (8)	-	-	1 (6)	-	-	-	-	-	-	-
Biochemical tests	121 (92)	71 (97)	23 (88)	37 (84)	11 (9)	10 (14)	5 (22)	9 (24)	1 (1)	1 (1)	1 (4)	2 (5)
Blood	97 (73)	43 (59)	16 (62)	28 (64)	9 (9)	8 (19)	5 (31)	8 (29)	-	1 (2)	1 (6)	2 (7)
Spinal fluid	87 (66)	62 (85)	16 (62)	23 (52)	1 (1)	-	-	3 (13)	-	-	-	-
Vitreous humor	32 (24)	32 (44)	7 (27)	8 (18)	3 (9)	1 (3)	-	1 (13)	1 (3)	-	-	-
Urine	26 (20)	30 (41)	12 (46)	21 (48)	-	-	-	2 (10)	-	-	-	-
Hair	6 (5)	3 (4)	2 (8)	1 (2)	-	-	-	-	-	-	-	-
Microbiological tests	127 (96)	73 (100)	25 (96)	40 (91)	28 (22)^*^	31 (42)	12 (48)	10 (25)	17 (13)	17 (23)	3 (12)	7 (18)
Blood	117 (89)	70 (96)	23 (88)	36 (82)	18 (15)	21 (30)	9 (39)	8 (22)	9 (8)	7 (10)	2 (9)	3 (8)
Spinal fluid	96 (73)	62 (85)	19 (73)	28 (64)	10 (10)	11 (18)	3 (16)	6 (21)	7 (7)	5 (8)	-	3 (11)
Throat/nasopharynx	122 (92)	73 (100)	23 (88)	35 (80)	22 (18)	18 (25)	8 (35)	8 (23)	-	-	-	-
Feces/rectum swab	87 (66)	50 (68)	19 (73)	17 (39)	8 (9)	10 (20)	1 (5)	-	1 (1)	5 (10)	1 (5)	-
Urine	45 (34)	37 (51)	14 (54)	22 (50)	2 (4)	1 (3)	1 (7)	2 (9)	-	-	-	-
Skin	17 (13)	15 (21)	5 (19)	5 (11)	-	2 (13)	-	1 (20)	-	-	-	1 (20)
Imaging	120 (91)	69 (95)	24 (92)	42 (95)	34 (28)^*^	34 (49)	16 (67)	19 (45)	17 (14)	5 (7)	9 (38)	8 (19)
CT scan	52 (39)	28 (38)	17 (65)	31 (70)	13 (25)	16 (57)	12 (71)	15 (48)	1 (2)	1 (4)	4 (24)	5 (16)
MRI scan	111 (84)	54 (74)	11 (42)	15 (34)	33 (30)	26 (48)	7 (64)	6 (40)	16 (14)	4 (7)	5 (45)	3 (20)
Autopsy	74 (56)	54 (74)	13 (50)	31 (70)	37 (50)*	42 (78)	10 (77)	26 (84)	27 (36)	33 (61)	6 (46)	17 (55)
Including brain	39 (30)	19 (26)	7 (27)	17 (39)	20 (51)	12 (63)	4 (57)	14 (82)	14 (36)	9 (47)	1 (14)	9 (53)
Metabolic testing	53 (40)	26 (36)	6 (23)	9 (20)	2 (4)	-	-	-	2 (4)	-	-	-
Genetic testing	51 (39)	28 (38)	8 (31)	19 (4)	8 (16)	2 (7)	1 (13)	4 (21)	2 (4)	1 (4)	1 (13)	2 (11)

^*^Significant difference (*p* < 0.025) compared to children ≥ 1 year old

**Table 4:** Use and yield of various diagnostics according to cause of death determined

Cause of death		Physical exam	Biochemical tests	Microbiological tests	Imaging	Autopsy	Metabolic testing	Genetic testing
Sepsis								
(*n* = 54)	Performed *N* (% total cases)	50 (93)	50 (93)	53 (98)	45 (83)	34 (63)	15 (28)	14 (26)
	Contributive *N* (% performed)	13 (26)	14 (28)	48 (91)	28 (62)	31 (91)	-	-
	Decisive *N* (% performed)	-	-	34 (64)	5 (11)	15 (44)	-	-
Cardiovascular								
(*n* = 50)	Performed *N* (% total cases)	44 (88)	44 (88)	50 (100)	49 (98)	42 (84)	14 (28)	32 (64)
	Contributive *N* (% performed)	2 (5)	3 (7)	10 (20)	19 (39)	38 (90)	-	11 (34)
	Decisive *N* (% performed)	-	1 (2)	-	5 (10)	36 (86)	-	5 (16)
Respiratory								
(*n* = 31)	Performed *N* (% total cases)	31 (100)	29 (94)	31 (100)	31 (100)	18 (58)	8 (26)	8 (26)
	Contributive *N* (% performed)	-	2 (7)	9 (29)	26 (84)	18 (100)	-	-
	Decisive *N* (% performed)	-	1 (4)	-	12 (39)	18 (100)	-	-
Gastrointestinal								
(*n* = 28)	Performed *N* (% total cases)	27 (96)	26 (93)	25 (89)	26 (93)	17 (61)	7 (25)	5 (18)
	Contributive *N* (% performed)	8 (30)	7 (27)	10 (40)	15 (58)	14 (82)	-	1 (20)
	Decisive *N* (% performed)	2 (7)	1 (4)	6 (24)	7 (27)	10 (59)	-	-
Neurological								
(*n* = 14)	Performed *N* (% total cases)	13 (93)	12 (86)	11 (79)	12 (86)	7 (50)	4 (29)	3 (21)
	Contributive *N* (% performed)	4 (31)	2 (17)	4 (36)	6 (50)	5 (71)	-	-
	Decisive *N* (% performed)	1 (8)	-	4 (36)	4 (33)	2 (29)	-	-
Other								
(*n* = 16)	Performed *N* (% total cases)	16 (100)	15 (94)	15 (94)	16 (100)	10 (59)	6 (38)	8 (50)
	Contributive *N* (% performed)	5 (31)	7 (47)	-	9 (56)	9 (90)	2 (33)	3 (38)
	Decisive *N* (% performed)	2 (13)	2 (13)	-	6 (38)	2 (20)	2 (33)	1 (13)

Numbers in decisive rows do not add up to total number since for some cases the history was decisive for determining the cause of death

## Discussion

This study is aimed at assessing the use and diagnostic value of the different postmortem diagnostic tests in the standardized PESUDIC procedure for determining the cause of death in sudden unexpected death in infants and children. In our cases of sudden and unexpected death of a child and a standard postmortem work-up, the cause of death was most often identified by autopsy, followed by microbiological testing. A median of three diagnostic modalities provided contributive findings per diagnosed case. History, imaging, and autopsy most often provided contributive results, although contributive findings were less frequent for infants.

In this study, autopsy provided a decisive cause of death; in almost half of the cases, it was performed, which corresponds to 43% of all cases with an explained or plausible cause of death. Therefore, 57% of all causes determined were identified through other investigations even though a significant percentage of these cases did have an autopsy performed. These results affirm the importance of an autopsy for sudden death cases in children but simultaneously highlight the value of performing a wide array of postmortem investigations. Cases that were diagnosed through the PESUDIC procedure had findings on a median of three investigations. Similarly, the study by Arnestad et al. reports that 67% of diagnoses in sudden deaths in children < 3 years were made through findings by two or more diagnostic investigations [4].

Whole-body imaging proved frequently contributive in our study. In the literature, postmortem MRI and CT are considered inferior to an autopsy for diagnosing macro-anatomic abnormalities [11, 14]. However, CT has proven to be specifically useful for identifying bony lesions and hemorrhages, whereas MRI has high diagnostic value for parenchymatous abnormalities such as organ lesions and infectious disease [14, 24]. Krentz et al. compared essential findings from autopsy and postmortem CT in 26 pediatric natural and traumatic deaths and found no significant differences between the methods [25]. Reportedly, per case, 1.0 essential finding was not visible at autopsy and 1.8 on CT. These studies therefore recommend a combination of postmortem whole-body imaging and autopsy for investigating pediatric cases [7, 14, 24, 25]. The results from our study support this conclusion.

Our findings show that microbiology is also an important test for a pediatric sudden death investigation. In our study, microbiological testing was both contributory and decisive in a considerable number of cases: 31% and 17%, respectively. Microbiology contributed to diagnosing the cause of death especially often for children between 1 and 12 years old and in cases that were ultimately diagnosed with a neurological or gastrointestinal infection or sepsis. In 96% of children, microbiological testing was performed, often at multiple sites. Contributive and decisive findings resulted from positive testing at multiple sites; therefore, postmortem microbiological testing should not be limited to blood culture alone. In previous studies, less children were diagnosed through microbiological testing as percentages of 2–8% were reported [4, 17, 26–28]. This could partly be explained by the routine use of microbiological testing in the PESUDIC procedure but also by the inclusion criteria of the procedure since children were allowed to have experienced prior symptoms such as a fever in contrast to other studies [29, 30]. Keeping the inclusion criteria for a thorough postmortem investigation broad allows for more causes of death to be determined.

Biochemical testing was less frequently useful for diagnosing PESUDIC cases. The results from biochemical testing in our PESUDIC cases were often difficult to interpret and muddled by the effects of decomposition and cell death. Literature on biochemical testing results in children is very limited, and no studies were found investigating the usefulness of different tests. Routine use of biochemical testing in these deaths is therefore not recommended, although it might be of use to rule out specific conditions such as hyperglycemia, electrolyte imbalance, or infection.

Infants less often had contributive findings from diagnostic tests in our study. A previous study on the PESUDIC outcomes has shown that 24% of infants were ultimately diagnosed with sudden infant death syndrome (SIDS) or unclassified sudden infant death (USID) according to the San Diego Classification [6]. This high incidence of SIDS within the PESUDIC infant cases could explain the lower yield of diagnostic tests for this age group since SIDS is characterized by a lack of abnormal findings, even though the pathology of SIDS is still unclear. However, SIDS is a diagnosis per exclusionem, warranting a thorough diagnostic evaluation, which affirms the need for a broad diagnostic protocol for all age groups.

Our study includes several strengths and limitations. Firstly, the study is based upon a large, national cohort of PESUDIC cases and therefore an unprecedented representation of sudden unexpected child deaths. Through the PESUDIC procedure, physical examination, biochemical analysis, microbiological testing, radiological imaging, and autopsy were routinely used. We are first to report on their separate and combined utility for investigating sudden death in infants and children. A limitation of our study due to its observational nature is that not all investigations were performed in all cases. However, more than 90% of cases had four or all investigations done. Another limitation is the fact that genetic testing, toxicological screening, and metabolic evaluation were only performed on specific indication, which might have led to missed diagnoses and biased results of this study. Lastly, we were unable to report on risk factors for SIDS cases since these were not systematically described in patient records.

The main purpose of postmortem investigations for infants and children with a sudden unexpected death is to find the cause of death and provide the families of these children with much-needed answers. These investigations are offered only once, and there are no second chances to be more thorough. In a postmortem setting, there is no option to await results from previous investigations before deciding on what testing should be done subsequently. Our findings show the important additional value of microbiological testing and whole-body imaging next to autopsy for finding the cause of death. Therefore, a routine protocol of postmortem investigations that includes autopsy, microbiological testing at multiple sites, and whole-body imaging at the minimum should be standard practice for cases of SUDI and SUDC.

## Data Availability

No datasets were generated or analysed during the current study.

## Abbreviations

CT: Computed tomography
MRI: Magnetic resonance imaging
PESUDIC: Postmortem evaluation of sudden unexpected death in infants and children
SUDI: Sudden unexpected death in infants
SUDC: Sudden unexpected death in children
